# Highly sensitive serological methods for detecting tomato yellow leaf curl virus in tomato plants and whiteflies

**DOI:** 10.1186/1743-422X-10-142

**Published:** 2013-05-06

**Authors:** Yan Xie, Xiaoyang Jiao, Xueping Zhou, Huan Liu, Yuequn Ni, Jianxiang Wu

**Affiliations:** 1 State Key Laboratory of Rice Biology, Institute of Biotechnology, Zhejiang University, Hangzhou, Zhejiang 310058, China

**Keywords:** Tomato yellow leaf curl virus, Whitefly, Monoclonal antibody, Dot enzyme-linked immunosorbent assay, Direct tissue blot immunoassay

## Abstract

**Background:**

Tomato yellow leaf curl virus (TYLCV) is a member of the genus *Begomovirus* in the family *Geminiviridae*, which causes severe losses in tomato production in tropic and subtropic regions.

**Methods:**

The purified TYLCV virions were used as the immunogen to produce monoclonal antibodies (MAbs) using the hybridoma technology. MAb-based dot enzyme-linked immunosorbent assay (dot-ELISA) and direct tissue blot immunoassay (DTBIA) were developed for sensitive, simple, and rapid detection of TYLCV in field tomato and whitefly (*Bemisia tabaci*) samples collected from TYLCV prevalent provinces in China.

**Results:**

Using the hybridoma technology, six murine MAbs (1C4, 8D10, 6E3, 2F2, 3F4 and 4G3) against TYLCV were prepared. Using the MAb 1C4, dot-ELISA and DTBIA were then established for detecting TYLCV in field tomato and whitefly samples collected from TYLCV prevalent provinces in China. The dot-ELISA could detect TYLCV in infected tissue crude extract diluted at 1:5,120 (w/v, g mL^-1^), and in viruliferous whitefly homogenate diluted at 1:128 (individual whitefly/μL), respectively. Field tomato samples (n=487) and whitefly samples (n=110) from TYLCV prevalent districts in China were screened for the presence of TYLCV using the two developed methods, and the results were further confirmed by PCR and nucleotide sequencing. The survey revealed that TYLCV is widespread on tomato plants in Zhejiang, Shandong and Henan provinces in China.

**Conclusions:**

The developed dot-ELISA is very suitable for the routine detection of TYLCV in field tomato and whitefly samples, and the DTBIA is more suitable for the routine detection of TYLCV in large-scale tomato plant samples collected from TYLCV prevalent areas.

## Background

Tomato yellow leaf curl disease (TYLCD) is one of the most significant viral diseases infecting tomato and causes severe economic losses worldwide [1]. It usually leads to yellow chlorosis on leaf margins and leaf curling symptoms, which results in yield loss and market value reduction. This disease is mainly caused by Tomato yellow leaf curl virus (TYLCV), which was first reported in Israel in 1964 [2], and later observed in several states in USA including Florida, Georgia, and Louisiana in 1990s [3-5]. In China, the first TYLCD-like disease was discovered in the suburbs of Shanghai in 2006 [6]. Recently TYLCV has outbroken in many districts in China and caused severe damage to tomato production [7]. Accurate detection and identification of this virus is the prerequisite for disease control.

TYLCV was first isolated and purified in 1988 [8]. TYLCV is a circular, single-stranded DNA (ssDNA) virus encapsidated in twinned quasi-isometric particles, containing a 2.7-2.8 kb genome. It consists of six open reading frames (ORFs) with two on the viral strand (V1 and V2) and four on the complementary strand (C1 to C4) [9,10]. TYLCV is transmitted by whiteflies (*Bemisia tabaci*) in a persistent manner at a high efficiency [11].

Plant virus in infected plants are usually determined by PCR analysis, which although is sensitive but not suitable for the routine field detection. Whereas, enzyme-linked immunosorbent assay (ELISA) has been become a routine method for detecting plant viruses in the past few decades. Since 1988, several serological detection methods based on polyclonal antibodies (PAbs) have been developed to detect TYLCV. However, the reported PAbs are only suitable for Western blot analysis rather than ELISA detection in field samples [12]. Recently, recombinant coat protein (CP) was used as an immunogen to produce MAbs and PAbs against TYLCV, and a TAS-ELISA was developed for detecting five begomoviruses [13]. In this study, six MAbs against TYLCV were produced using the hybridoma technology and two serological methods, dot enzyme-linked immunosorbent assay (dot-ELISA) and direct tissue blot immunoassay (DTBIA) using the most sensitive MAb 1C4 were successfully developed for detecting TYLCV in field plant and insect vector samples. The survey of the field samples with the dot-ELISA and DTBIA demonstrated that TYLCV is widespread in fields in Zhejiang, Shandong and Henan provinces.

## Results

### Virus purification

**Figure 1:**
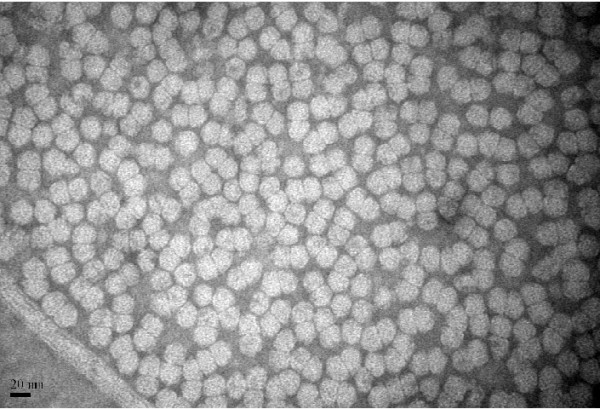
**Electron micrograph of purified Tomato yellow leaf curl virus.** Bar= 20 nm.

TYLCV particles were purified by differential centrifugation and examined by transmission electron microscopy. Twinned and icosahedral virions about 20 nm in diameter were observed in the purified preparation, which were the typical morphology of virus particles in the genus *Begomovirus* (Figure 1).

### Production and characterization of MAbs against TYLCV

The purified TYLCV virions were used as the immunogen and injected into six BALB/c mice. After the 4^th^ immunization, the spleen cells of the immunized mice were taken out to use for hybridoma preparation. Via cell fusion, cell culture, antibody detection and cell cloning, six hybridoma lines (1C4, 8D10, 6E3, 2F2, 3F4 and 4G3) secreting MAbs against TYLCV were obtained and injected intraperitoneally into pristine-primed BALB/c mice to produce ascitic fluids, respectively. The immunoglobulin classes and subclasses of the four MAbs (1C4, 2F2, 3F4 and 4G3) were isotyped as IgG1, while other two MAbs (8D10 and 6E3) were isotyped as IgG2a (Table 1). The light chains of the six MAbs were of the kappa light chain type (Table 1). The IgG yields of MAbs from ascitic fluids ranged from 2.01 to 9.23 mg mL^-1^. The titers of six MAbs in ascites determined by an indirect-ELISA ranged from 10^-6^ to 10^-7^ (Table 1).

**Table 1:** Properties of monoclonal antibodies against TYLCV

**MAbs**	**Isotype**	**Ascites titre**	**IgG yield (mg mL**^**-1**^**)**
1C4	IgG1, κ chain	10^-7 a^	4.11
8D10	IgG2a, κ chain	10^-7^	2.01
6E3	IgG2a, κ chain	10^-6^	5.05
2F2	IgG1, κ chain	10^-7^	4.99
3F4	IgG1, κ chain	10^-6^	9.23
4G3	IgG1, κ chain	10^-7^	3.34

^a^ The MAb titer was the last dilution that yielded an absorption value above 0.3 at 30 min after adding the substrate at room temperature.

**Figure 2:**
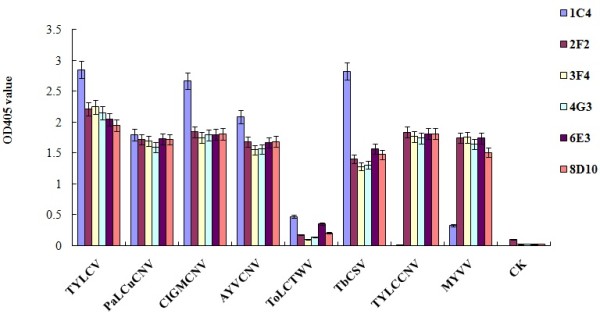
**Specificity analyses of six MAbs by TAS-ELISA.** The OD405 value was the mean value obtained from three independent assays at 30 min after adding the substrate at room temperature. Leaf tissues extracts were diluted at 1:30 (w/v, g mL^-1^) in PBS. CK- denoted the healthy plant tissues.

The reactions of six MAbs with eight begomoviruses, TYLCV, Papaya leaf curl China virus (PaLCuCNV), Clerodendrum golden mosaic China virus (CIGMCNV), Ageratum yellow vein China virus (AYVCNV), Tomato leaf curl Taiwan virus (ToLCTWV), Tobacco curly shoot virus (TbCSV), Tomato yellow leaf curl China virus (TYLCCNV) and Malvastrum yellow vein virus (MYVV) were determined by a triple antibody sandwich enzyme-linked immunosorbent assay (TAS-ELISA) as described previously [14]. The results showed that the five MAbs except 1C4 could react strongly with TYLCV-, PaLCuCNV-, CIGMCNV-, AYVCNV-, TbCSV-, TYLCCNV- and MYVV-infected plant tissues, weakly with ToLCTWV-infected plant tissues, but not with healthy plant tissues. Compared with other five MAbs, 1C4 also could react strongly with TYLCV-, PaLCuCNV-, CIGMCNV-, AYVCNV- and TbCSV-infected plant tissues, mildly with MYVV and ToLCTWV, but not with TYLCCNV and healthy plant tissues (Figure 2).

**Figure 3:**
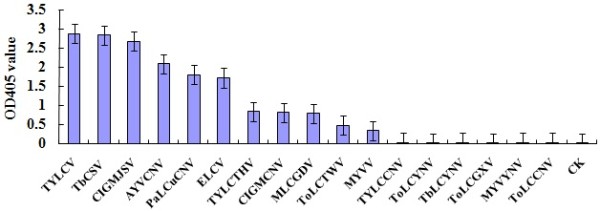
**ACP-ELISA results of the MAb 1C4 with 17 different begomoviruses.** The OD405 value was the mean value obtained from three samples at 30 min after adding the substrate at room temperature. Leaf extracts were diluted at 1:30 (w/v, g mL^-1^) in 0.05 mol L^-1^ sodium bicarbonate buffer. CK- was the healthy plant tissues.

To understand the broad-specificity of the MAb 1C4 for begomoviruses, 17 begomoviruses, TYLCV, PaLCuCNV, ClGMCNV, AYVCNV, ToLCTWV, TbCSV, TYLCCNV, MYVV, Tomato leaf curl China virus (ToLCCNV), Tomato leaf curl Guangxi virus (ToLCGXV), Tomato leaf curl Yunnan virus (ToLCYNV), Tobacco leaf curl Yunnan virus (TbLCYNV), Tomato yellow leaf curl Thailand virus (TYLCTHV), Malvastrum yellow vein Yunnan virus (MYVYNV), Malvastrum leaf curl Guangdong virus (MLCGDV), Euphorbia leaf curl virus (ELCV) and Clerodendrum golden mosaic Jiangsu virus (ClGMJSV) were further tested by antigen-coated plate enzyme-linked immunosorbent assay (ACP-ELISA) as described previously [14]. The detection results demonstrated that MAb 1C4 reacted strongly with TYLCV-, TbCSV-, CIGMJSV-, AYVCNV-, PaLCuCNV- and ELCV-infected plant tissues, mildly with TYLCTHV-, CIGMCNV-, MLCGDV-, ToLCTWV- and MYVV- infected plant tissues, but not with TYLCCNV-, ToLCYNV-, TbLCYNV-, ToLCGXV-, MYVYNV-, ToLCCNV-infected and healthy plant tissues (Figure 3).

**Figure 4:**
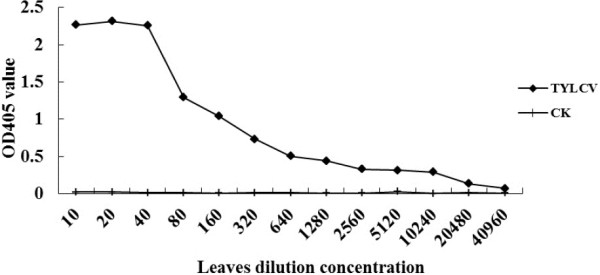
**Sensitivity analysis of the MAb 1C4 by ACP-ELISA.** Crude extracts from a TYLCV-infected tomato plant and a healthy tomato plant(CK-) were serial two-fold diluted in 0.05 mol L^-1^ sodium bicarbonate buffer from 1:10 to 1:40960 (w/v, g mL^-1^) and used as coating antigens, respectively. The OD405 value was the mean value obtained from three independent assays at 30 min after adding the substrate at room temperature. The dilution endpoint of ACP-ELISA was 1:10,240 (w/v, g mL^-1^).

The sensitivity of the MAb 1C4 for detecting TYLCV was also analyzed by ACP-ELISA. The crude extract from TYLCV-infected plant tissues was serial two-fold diluted from 1:10 to 1:40,960. The analytic results indicated that MAb 1C4 could detect TYLCV in infected plant tissue crude extract diluted at 1:10,240 (w/v, g mL^-1^) (Figure 4). Therefore, MAb 1C4 was highly sensitive for TYLCV detection.

### DTBIA for TYLCV detection in tomato plants

The working dilutions of the MAb 1C4 and the goat anti-mouse IgG conjugated with alkaline phosphatase (AP) (Sigma-Aldrich, St. Louis, MO, USA) were determined by phalanx tests [14]. The results of the three independent DTBIA revealed that TYLCV was readily detected in infected plant tissues when the MAb and the goat anti-mouse IgG conjugated with AP were used at the dilutions of 1:5,000 and 1:8,000, respectively.

**Figure 5:**
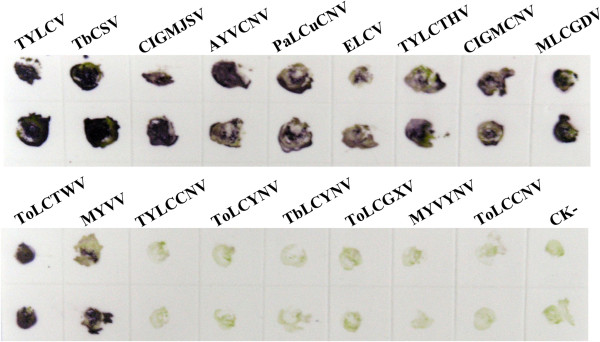
**Broad-specificity analysis of the DTBIA for detecting begomoviruses.** Upper and lower two dots were repeats of the same sample.

Using the MAb 1C4, the DTBIA had positive reactions of detection not only with TYLCV, but also with TbCSV, CIGMJSV, AYVCNV, PaLCuCNV, ELCV, TYLCTHV, CIGMCNV, MLCGDV, ToLCTWV and MYVV in their infected plants, but negative reactions were obtained with TYLCCNV, ToLCYNV, TbLCYNV, ToLCGXV, MYVYNV or ToLCCNV-infected plants or healthy plant tissues (Figure 5). Those results suggest that besides detecting TYLCV in tomato plants, the DTBIA can also detect TbCSV, CIGMJSV, AYVCNV, PaLCuCNV, ELCV, TYLCTHV, CIGMCNV, MLCGDV, ToLCTWV, or MYVV in their infected plants in their prevalent areas.

### Dot-ELISA for TYLCV detection in plant samples

The results of the three independent phalanx tests demonstrated that TYLCV was readily detected in infected plant tissues by dot-ELISA when the MAb and the goat anti-mouse IgG conjugated with AP (Sigma-Aldrich) were used at the dilutions of 1:5,000 and 1:8,000, respectively.

**Figure 6:**
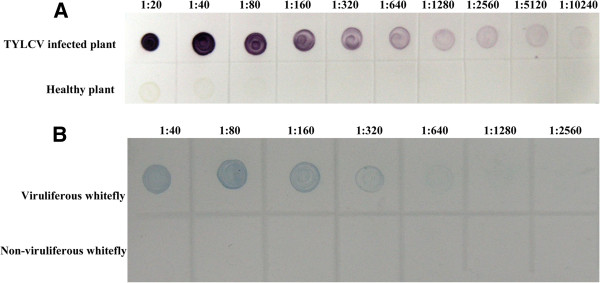
**Sensitivity analyses of the dot-ELISA for plant (A) and vector (B) detection. A**: Sensitivity analysis of the dot-ELISA for plant detection. TYLCV-infected and the healthy (CK-) leaf extracts were two-fold diluted with PBS. The dilution endpoint of dot-ELISA was 1:5,120 (w/v, g mL^-1^). **B**: Sensitivity analysis of the dot-ELISA for vector detection. Viruliferous and the non-viruliferous whitefly homogenates were two-fold diluted in with PBS, respectively. The dilution endpoint of dot-ELISA for vector detection was 1:128 (individual whitefly/μL).

**Figure 7:**
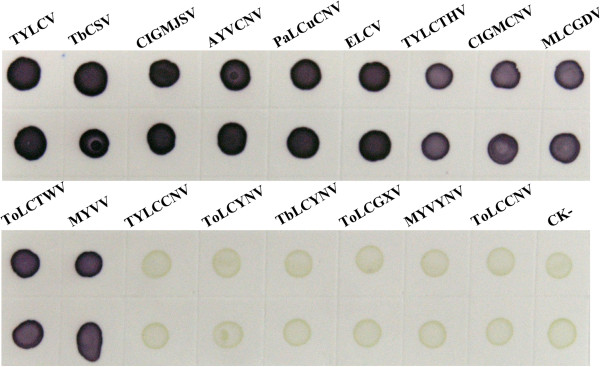
**Broad-specificity analysis of the dot-ELISA for detecting begomoviruses.** Upper and lower two dots were repeats of the same sample.

Serial two-fold dilutions with PBS of TYLCV-infected tomato plants were used to determine the sensitivity of the dot-ELISA. The results showed that dot-ELISA could detect TYLCV in infected tissue crude extract diluted at 1:5120 (w/v, g mL^-1^) (Figure 6A), which indicated that this assay was highly sensitive for TYLCV detection in tomato plant samples. To analyze the broad-specificity of the dot-ELISA for detecting begomoviruses, the crude extracts from a healthy plant and TYLCV-, TbCSV-, CIGMJSV-, AYVCNV-, PaLCuCNV-, ELCV-, TYLCTHV-, CIGMCNV-, MLCGDV-, ToLCTWV-, MYVV-, TYLCCNV-, ToLCYNV-, TbLCYNV-, ToLCGXV-, MYVYNV- and ToLCCNV-infected plants were respectively spotted onto nitrocellulose membranes and detected for the virus by the dot-ELISA. The results demonstrate that the dot-ELISA can be used to detect TYLCV, TbCSV, CIGMJSV, AYVCNV, PaLCuCNV, ELCV, TYLCTHV, CIGMCNV, MLCGDV, ToLCTWV and MYVV in their infected plants, but can not be used to detect TYLCCNV, ToLCYNV, TbLCYNV, ToLCGXV, MYVYNV and ToLCCNV in their infected plants (Figure 7). The result suggests that besides detecting TYLCV in tomato plants, the dot-ELISA can also detect TbCSV, CIGMJSV, AYVCNV, PaLCuCNV, ELCV, TYLCTHV, CIGMCNV, MLCGDV, ToLCTWV or MYVV in their infected plants in their prevalent areas.

### Dot-ELISA for TYLCV detection in field whitefly samples

The results of three independent phalanx tests showed that the dilutions of the MAb 1C4 at 1:3,000 and the goat anti-mouse IgG conjugated with horseradish peroxidase (HRP) (Sigma-Aldrich) at 1:5,000 were optimal for dot-ELISA to detect TYLCV in whitefly samples. Under this optimal condition, dot-ELISA could detect TYLCV in an individual whitefly homogenate diluted at 1:128 (individual whitefly/μL) (Figure 6B).

**Figure 8:**
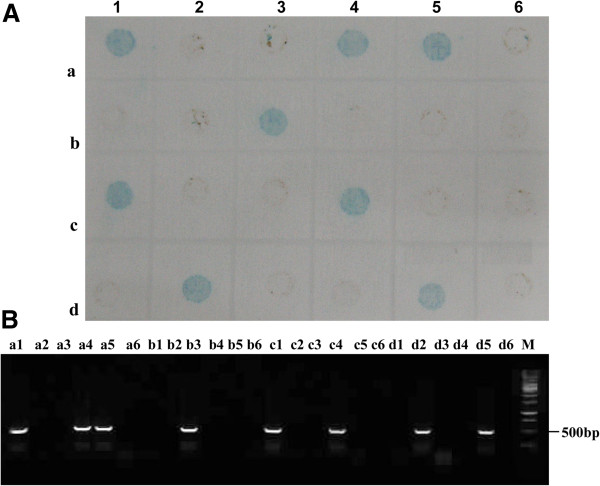
**The detection results of field whitefly samples by dot-ELISA (A) and PCR (B). A**: The detection result of field whitefly samples by dot-ELISA. a1-6, b1-6, c1-6 and d1-4 were 22 of 110 field whitefly samples. d5 and d6 were the positive and negative controls. **B**: The detection result of field whitefly samples by PCR. The samples were same as those described in Figure 8A. Lane M was 1 kb DNA marker.

The 110 whiteflies collected from TYLCV-infected tomato fields in Zhejiang, Shandong and Henan provinces in China were detected for TYLCV by the developed dot-ELISA and the representative results were shown in Figure 8A. Among 110 whiteflies, 39 samples were tested positive by the dot-ELISA (Table 2). All these whitefly samples were simultaneously analyzed by PCR and nucleotide sequencing and the results of PCR detection and nucleotide sequencing were in accordance with that of dot-ELISA (Figure 8B; Table 2). This confirmed that the developed dot-ELISA was an effective method for TYLCV detection in whitefly vectors collected from only TYLCV prevalent areas.

**Table 2:** Detection of TYLCV in field samples by dot-ELISA, DTBIA and PCR

**Sample sources**	**TYLCV positive No./tomato sample No.**	**TYLCV positive No./whiltefly No.**
Henan Province	6,6,6/9^a^	7,7 /22 ^b^
Shandong Province	30,30,30/56	11,11/20
Zhejiang Province	195,195,197 /422	21,21/68
Total	231, 231,233 /487	39,39/110

^a^ 6, 6, 6/9 represent 6, 6, 6 of 9 samples were TYLCV-positive by dot-ELISA , DTBIA and PCR, respectively.

^b^ 7,7/22 represent 7, 7 of 22 whitefly samples were TYLCV-positive by dot-ELISA and PCR, respectively.

### Detection of field plant samples by the DTBIA and dot-ELISA

**Figure 9:**
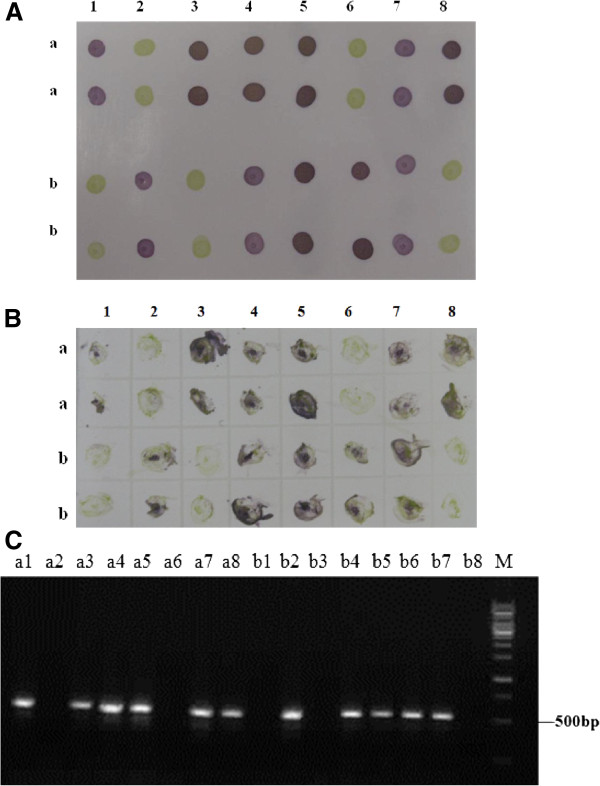
**The detection results of field tomato plant samples by dot-ELISA (A), DTBIA (B) and PCR (C). A**: The detection results of field tomato plant samples by dot-ELISA. a1-a8, b1-b6 were field samples, b7 and b8 were positive and negative controls, respectively. Upper and lower two dots were repeats of the same sample. **B**: The detection results of field tomato plant samples by DTBIA. The samples were same as those described in Figure 9A. Upper and lower two dots were repeats of the same sample. **C**: The detection results of field tomato plant samples by PCR. The samples were same as those described in Figure 9A. Lane M was 1kb DNA marker.

A total of 487 field tomato samples showing virus-like symptoms from TYLCV prevalent Zhejiang, Shandong and Henan provinces in China were screened the presence of TYLCV using the developed dot-ELISA and DTBIA. The results showed that 233 of 487 tomato samples were tested positive by the dot-ELISA and DTBIA (Figure 9A, B; Table 2). All 487 field tomato plant samples were further detected for TYLCV by PCR using the degenerated primer pair PA/PB (Figure 9C), and the PCR products were cloned and sequenced. The amplified nucleotide sequences of Chinese isolates were compared with the TYLCV sequences in GenBank, and the results demonstrated that amplified products shared more than 97.6% identity with the TYLCV sequences in GenBank. The results of PCR and nuclutide sequencing further confirmed that the detection results of the dot-ELISA and DTBIA. These results suggested that the dot-ELISA and DTBIA could be used to detect TYLCV in field tomato plants collected form TYLCV prevalent areas in China, and TYLCV is widespread in Zhejiang, Shandong and Henan provinces.

## Discussion

In the process of this study, we have selected more than 60 MAbs, but have not selected a TYLCV-specific MAb (data not shown). Based on this result and the conservation of amino acid sequences of coat proteins of begomoviruses, we assume that it is difficult to produce TYLCV-specific antibodies. However, we will further prepare a TYLCV-specific MAb for the specific detection of TYLCV. At present, field tomato plants in Zhejiang, Shanghai, Jiangsu, Anhui, Shandong, Sichuan, Henan, Hebei, Liaoning, Xinjiang, Inner Mongolia, Shanxi and Beijing in China are only infected by TYLCV, while in Yunnan, Guangxi, Guangdong and Hainan in China are infected by TYLCV, TYLCTHV, TYLCCNV, ToLCCNV, ToLCGXV, ToLCYNV and PaLCuCNV ( to be published data). Although the two developed serological assays based on the MAb 1C4 can detect not only TYLCV, but also TYLCTHV, ToLCTWV and PaLCuCNV, which infects tomato plants, we assume that the two serological assays can detect the infection of TYLCV on tomato plants in only TYLCV prevalent areas in most parts of China. At the same time, the two serological assays can be used to detect other begomoviruses, i.e. TbCSV, CIGMJSV, AYVCNV, PaLCuCNV, ELCV, TYLCTHV, CIGMCNV, MLCGDV, ToLCTWV and MYVV in their infected plants in their epidemic areas.

Recently, TYLCV and other begomoviruses are primarily identified by PCR amplification, which is time-consuming, complex, expensive and dependent on instruments. In this study, six sensitive MAbs were prepared using purified TYLCV particles as the immunogen and two serological methods (dot-ELISA and DTBIA) were successfully developed and applied to detect TYLCV in field tomato plants and whitefly vectors collected from TYLCV prevalent areas in China. The newly developed dot-ELISA could detect TYLCV in infected tomato plant tissue extracts diluted at 1:5,120 (w/v, g mL^-1^), and in an individual viruliferous whitefly homogenate diluted at 1:128 (individual whitefly/μL), respectively. To our knowledge, this is the first study on detecting TYLCV with dot-ELISA and DTBIA in tomato plants and especially in whitefly vectors.

The dot-ELISA and DTBIA are simple, quick, economical and particularly attractive for high-throughput detection of TYLCV in field plant and whitefly samples. Furthermore, the results of dot-ELISA and DTBIA can be easily interpreted with the naked eyes. Therefore, these two methods can be used as routine diagnostic methods to study etiology and epidemiology of TYLCV in TYLCV prevalent areas in China, and significantly promote the detection technology of TYLCV. Additionally, the wide application of these two serological methods for TYLCV detection can serve in diagnosis, forecasting and scientific controls of TYLCD in TYLCV prevalent fields in China.

## Conclusions

Both two developed dot-ELISA and DTBIA are suitable for sensitive, rapid and high- throughput detection of TYLCV in field tomato plants collected from TYLCV prevalent areas, and the dot-ELISA is very suitable for the routine TYLCV detection of large-scale whitefly vectors in TYLCV prevalent areas in most parts of China. The field survey results demonstrated that TYLCV is widespread in Zhejiang, Shandong and Henan provinces.

## Materials and methods

### Plant and whitefly samples and virus sources

In 2012, tomato plants showing virus-like symptoms were collected from TYLCV prevalent areas, i.e. Hangzhou and Wenzhou in Zhejiang Province, Zhengzhou in Henan Province, Weifang in Shandong Province. Whitefly samples were collected from the TYLCV-infected tomato fields in Zhejiang, Shandong and Hennan provinces, and kept in 95% ethanol. The TYLCV-infected tomato plants and viruliferous whiteflies fed on TYLCV-infected tomato plants were used as positive controls, and healthy tomato plants and the non-viruliferous whiteflies were used as negative controls.

TYLCV, PaLCuCNV, TbCSV, AYVCNV, ToLCTWV, ToLCCNV, ToLCGXV, ToLCYNV, TbLCYNV, TYLCTHV, MYVV, MYVYNV, MLCGDV, ELCV, CIGMCNV, ClGMJSV and TYLCCNV were previously identified by the author’s laboratory and maintained on *Nicotiana benthamiana* or *Solanum lycopersicum* plants by agro-inoculation with viral infectious clones in an insect-proof greenhouse.

### Mice and animal experiments

Animal experiments were carried out using female BALB/c mice provided by the Shanghai Laboratory Animal Center of the Chinese Academy of Sciences (Certificate of animal quality: Zhong Ke Dong Guan No.003) at the Research Center of the Laboratory of Animal Science, Zhejiang College of Traditional Chinese Medicine, Hangzhou, China. The animal experiments were performed according to the Principles of the Helsinki accord. The experimental protocols were approved by the Animal Ethics Committee of Zhejiang University, Hangzhou, China.

### Preparation of MAbs against TYLCV

TYLCV particles were purified from 250 g infected tissues of *N. benthamiana* agro-inoculated by TYLCV infectious clone as described by Czosnek et al. [12] and used as the immunogen. The purified virions were stained with 2% (w/v, g mL^-1^) phosphotungstic acid and examined with an electron microscope (JEM−1200 EX, JEOL Ltd., Tokyo, Japan). Preparation of MAb against TYLCV was performed using the protocol as described previously [13].

### TAS-ELISA and ACP-ELISA

TAS-ELISA and ACP-ELISA were carried out by following the standard procedures described previously by Shang et al. [14]. Samples are considered to be positive when absorbance values are at least three times greater than the negative controls.

### Dot-ELISA for TYLCV detection in plant and whitefly samples

The dot-ELISA procedures were performed according to the method described previously with slight modification [14]. Briefly, plant samples were ground with a mortar and pestle in 0.01 mol L^-1^ phosphate buffered saline (PBS, pH 7.4) (1 g plant tissue in 10 mL PBS), and then centrifuged at 5000×g for 3 min. An individual whitefly was placed in 2 μL PBS on a piece of parafilm membrane and ground with the bottom of a 0.5 mL eppendorf centrifuge tube. The supernatants of plant samples and the homogenates of whiteflies were respectively spotted onto nitrocellulose membranes (Amersham Biosciences, Bucks, UK, 2 μL/spot) and allowed to be air-dried at room temperature for 10 min. Negative and positive controls were spotted with extracts from healthy and TYLCV-infected plant tissues or the homogenate of non-viruliferous and viruliferous whiteflies, respectively. After blocked with 5% skimmed milk for 30 minutes, the membranes were incubated in suitably diluted MAb at 37°C for 1 h. After four time washes with PBST (0.01 mol L^-1^ PBS containing 0.05% Tween-20, pH 7.4), the membranes were incubated in suitably diluted goat anti-mouse IgG conjugated with AP for plant samples or HRP (Sigma-Aldrich) for whitefly samples at 37°C for another 1 h. Finally, after five time washes with PBST, the membranes were color-developed in NBT/BCIP (5-bromo-4-chloro-3-indolyl phosphate/nitro–blue tetrazolium chloride) or TMB (3, 3’, 5, 5’-tetramethylbenzidine) substrate solution (Promega, Madison, WI, USA) for the AP and HRP conjugates, respectively. Positive samples developed either purple or blue color during 10–25 min.

### DTBIA for TYLCV detection in plant samples

The DTBIA procedures were operated as described previously [14]. Briefly, stems of the tested plants were transversely cut with a surgical blade, and the cross sections were pressed onto a nitrocellulose membrane for 3–5 sec. Negative and positive controls were healthy and TYLCV-infected plants, respectively. The tissue blots were air-dried at room temperature for 10 min. The following steps of DTBIA were the same as that of the dot-ELISA.

### PCR analysis and sequencing

The total DNA from plant samples was extracted using the CTAB method as described by Xie *et al.*[15] and whitefly DNA was extracted as described by De Barro and Driver [16]. The degenerated primer pair PA (5’-TAATATTACCKGWKGVCCSC-3’) and PB (5’-TGGACYTTRCAWGGBCCTTCACA-3’) (B=C, T or G, K=G or T, R=A or G, S=C or G, V=A, C or G, W=A or T, Y= C or T) were used to amplify an approximately 500 bp fragment covering a portion of the intergenic region (IR) and the *AV1* gene of the DNA as described by Xie et al. [15]. The amplified fragments were cloned and sequenced using the automated model 3730 DNA sequencing system (Perkin Elmer, Foster City, CA, USA).

## Competing interests

The authors declare that they have no competing interests.

## Authors’ contributions

YX, XJ, HL and YN performed the experiments. YX, XJ, JW involved in data analysis and manuscript preparation. JW, XZ provided overall direction and conducted experimental design. All authors read and approved the final manuscript.
